# Differentiation impairs Bach1 dependent HO-1 activation and increases sensitivity to oxidative stress in SH-SY5Y neuroblastoma cells

**DOI:** 10.1038/s41598-017-08095-7

**Published:** 2017-08-08

**Authors:** Sabrina Piras, Anna Lisa Furfaro, Lorenzo Brondolo, Mario Passalacqua, Umberto Maria Marinari, Maria Adelaide Pronzato, Mariapaola Nitti

**Affiliations:** 1 Department of Experimental Medicine, University of Genoa, Via L.B. Alberti 2, 16132 Genoa, Italy; 2 Giannina Gaslini Institute, Via Gerolamo Gaslini 5, 16147 Genoa, Italy

## Abstract

Neuronal adaptation to oxidative stress is crucially important in order to prevent degenerative diseases. The role played by the Nrf2/HO-1 system in favoring cell survival of neuroblastoma (NB) cells exposed to hydrogen peroxide (H_2_O_2_) has been investigated using undifferentiated or all-trans retinoic acid (ATRA) differentiated SH-SY5Y cells. While undifferentiated cells were basically resistant to the oxidative stimulus, ATRA treatment progressively decreased cell viability in response to H_2_O_2_. HO-1 silencing decreased undifferentiated cell viability when exposed to H_2_O_2_, proving the role of HO-1 in cell survival. Conversely, ATRA differentiated cells exposed to H_2_O_2_ showed a significantly lower induction of HO-1, and only the supplementation with low doses of bilirubin (0,5–1 μM) restored viability. Moreover, the nuclear level of Bach1, repressor of HO-1 transcription, strongly decreased in undifferentiated cells exposed to oxidative stress, while did not change in ATRA differentiated cells. Furthermore, Bach1 was displaced from HO-1 promoter in undifferentiated cells exposed to H_2_O_2_, enabling the binding of Nrf2. On the contrary, in ATRA differentiated cells treated with H_2_O_2_, Bach1 displacement was impaired, preventing Nrf2 binding and limiting HO-1 transcription. In conclusion, our findings highlight the central role of Bach1 in HO-1-dependent neuronal response to oxidative stress.

## Introduction

Cell ability to adapt to stressful conditions is crucial to maintain physiological functions over time. While a severe imbalance between oxidative insults and antioxidant defenses leads to cell damage and death, in presence of functional antioxidants different redox-dependent signaling pathways can be modulated by low amount of reactive oxygen species (ROS), leading to different cell responses, from differentiation to proliferation^1, 2^.

Due to the high rate of ROS generation, the high content of lipids susceptible to peroxidation, and the relatively low amount of antioxidant defenses, neuronal cells are especially sensitive to oxidative damage in comparison to other cell types^3^. However, ROS can act as signaling molecules in neuronal cells too, for instance, as far as the differentiation activity of retinoic acid is concerned^4–6^. Thus, the ability to balance oxidative insults is crucial for neuronal cell survival.

Among the inducible antioxidant defenses heme oxygenase 1 (HO-1) plays a key role^7^. Indeed, HO-1 is the inducible form of HO system, which carries out the degradation of the iron-containing molecule heme and generates free iron (Fe^2+^), carbon monoxide and biliverdin. Free iron is quickly quenched by ferritin, which is synthesized in parallel with HO-1 induction^8^, and biliverdin is further converted into bilirubin by the activity of biliverdin reductase^9^. Overall ferritin, carbon monoxide and bilirubin exert strong antioxidant, antiapoptotic and anti-inflammatory activities^8, 10–12^.

HO-1 transcription is induced by multiple redox dependent-signaling pathways such as MAPK, PI3K/AKT kinases, STAT3, AP-1 and especially by the nuclear factor erythroid 2-related factor 2 (Nrf2)^13^. Nfr2, indeed, drives the adaptive responses of cells under electrophylic or oxidative stimuli. Under stressed conditions, it is released from its negative regulator Kelch-like ECH-associated protein 1 (Keap-1) and moves from the cytosol into the nucleus^14^. The binding to the Antioxidant Response Element (ARE) sequences in the promoter region of target genes enables the transcription of a plethora of antioxidant and protective genes^15, 16^.

However, a few number of repressors of HO-1 transcription have been identified, namely Keap1 which favors Nrf2 proteasomal degradation in unstressed conditions^17^, and Bach1 which prevents Nrf2 binding to the ARE sequences^18^. Moreover, Bach1 is directly involved in heme homeostasis thus playing a specific role in the induction of HO-1^19^.

We previously showed that retinoic acid-induced neuroblastoma (NB) differentiation increases the generation of anion peroxide from the coordinated activation of PKC delta and NADPH oxidase favoring neurite elongation^5^. However, we also provided evidence that, after retinoic acid induced differentiation, cells become more sensitive to the oxidative stress induced by advanced glycation end-products (AGEs)^20^.

In this work we show that NB cell differentiation induced by retinoic acid modifies the activation of Nrf2 and HO-1, impairing the ability to counteract oxidative stress.

## Results

### ATRA-differentiated cells are more sensitive to H_2_O_2_ than undifferentiated ones

**Figure 1:**
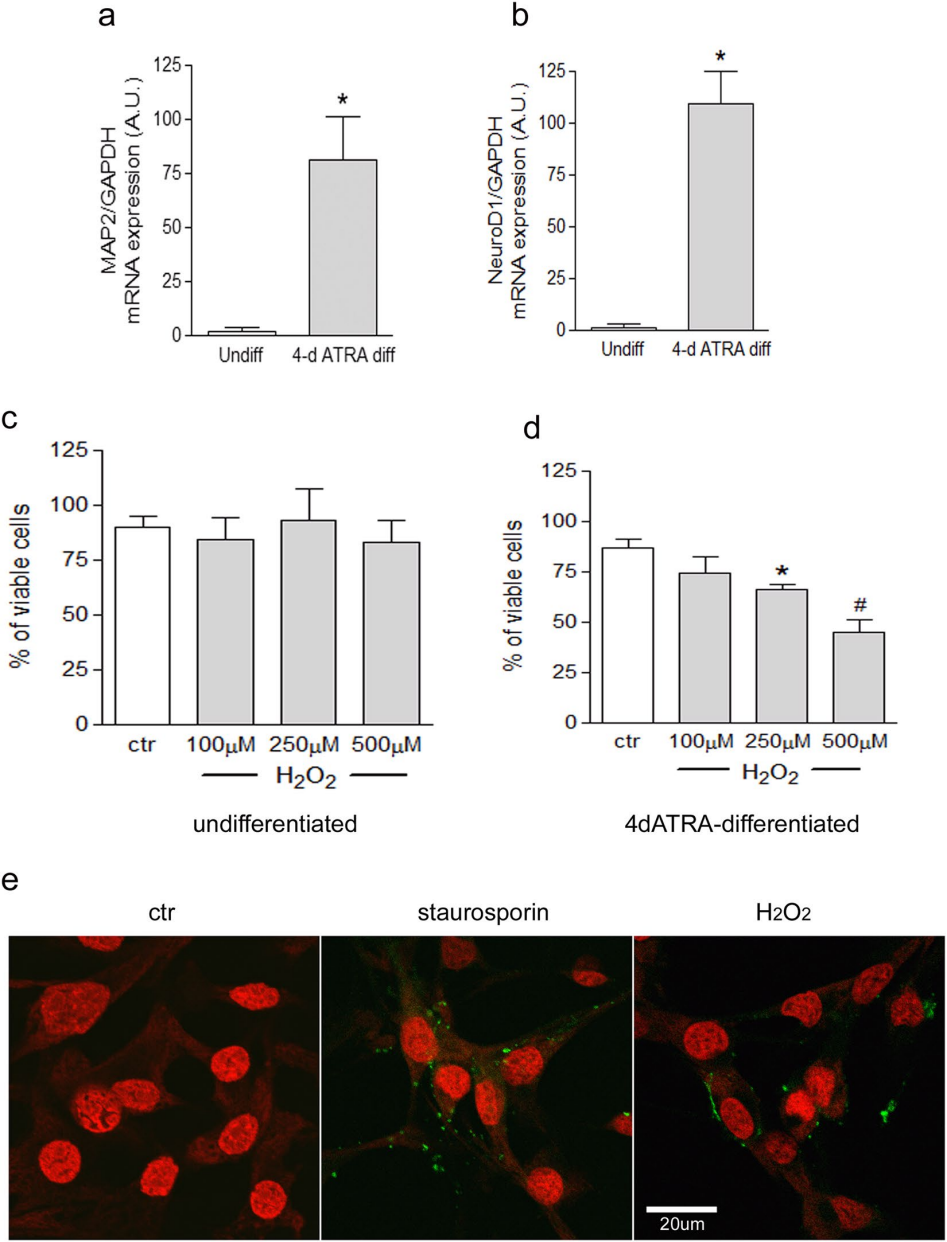
ATRA-induced differentiation increases sensitivity to H_2_O_2_, favoring the onset of apoptosis. (**a** and **b**) Cell differentiation is checked by RT-PCR analysis of MAP2 and NeuroD1. Statistical analysis: n = 3, *p < 0.05 vs undiff. (**c** and **d**) The number of viable cells have been analyzed by using Trypan blue dye after 24 h exposure to H_2_O_2_ and expressed as a percentage of viable cells. Statistical analysis: n = 4, *p < 0.05 and ^#^p < 0.01 vs control cells. (**e**) Positivity to Annexin V-FITC (green staining) of 4d-ATRA differentiated cells has been checked as a marker of early apoptosis after 24 h treatment with 500 µM H_2_O_2_ and appears as a spotted green membrane fluorescence. Treatment with 100 nM staurosporin has been used as positive control. Nuclei are counterstained by To-Pro3 iodide as detailed in Materials and Methods. Scale bar = 20 µm.

The effect of 24 h exposure to increasing concentrations of H_2_O_2_ (from 100 μM to 500 μM) on undifferentiated or differentiated SH-SY5Y neuroblastoma (NB) cell viability has been tested. In previous papers we showed that cell differentiation with all-trans retinoic acid for 4 or 7 days (4d-ATRA and 7d-ATRA) increases the number and the length of neurites, slows down the cell cycle and increases the expression of MAP2 as neurite marker^5, 21^. In the present work, the up-regulation of MAP2 and NeuroD1^22^ have been routinely checked by using RT-PCR to confirm differentiation (Fig. 1a and b).

The mean value of viability of untreated undifferentiated cells was 90% and no modifications were induced by H_2_O_2_ treatments (Fig. 1c). The mean value of viability of untreated 4d-ATRA differentiated cells was 86% and was reduced to 66% and 45% after cell exposure to 250 μM and 500 μM H_2_O_2_, respectively (Fig. 1d). Moreover, confocal microscopy analysis of Annexin V positivity showed that 4d-ATRA differentiated cells exposed to 500 μM H_2_O_2_ increased the membrane expression of phosphatidylserine. The same pattern of expression has been observed in 4d-ATRA differentiated cells treated with staurosporin used as positive control of early apoptosis. On the contrary, untreated cells did not show any membrane staining (Fig. 1e).

When 7d-ATRA differentiated cells have been used, the number of viable cells was further decreased to 57% and 44% by the exposure to 250 μM and 500 μM H_2_O_2_ (Supplementary Fig. 1). However, in the following experiments, a single dose of 500 μM H_2_O_2_ has been used on undifferentiated and 4d-ATRA differentiated cells.

### HO-1 mRNA is differently expressed in undifferentiated and differentiated cells treated with H_2_O_2_

**Figure 2:**
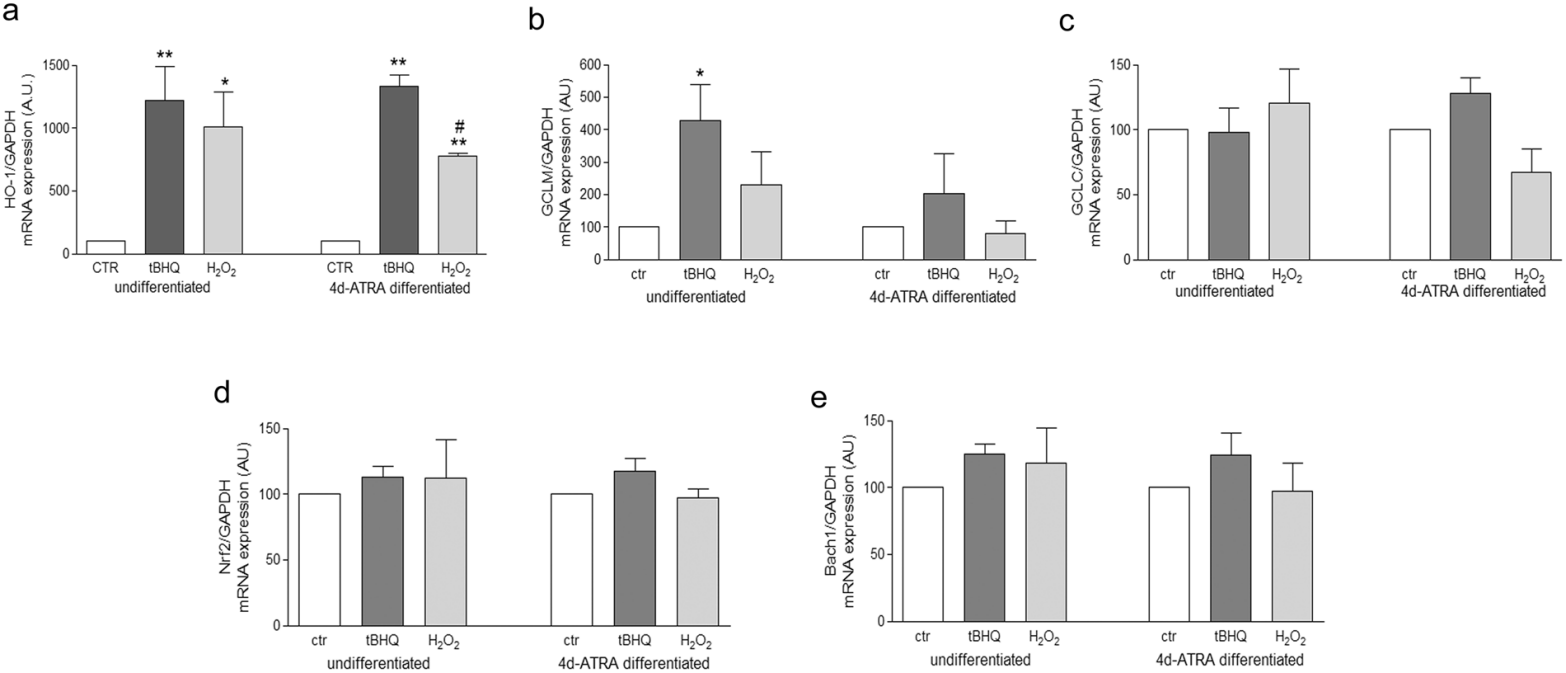
HO-1 mRNA expression is differently regulated in response to oxidative stress before and after cell differentiation. RT-PCR analysis of HO-1(**a**), GCLM (**b**) and GCLC (**c**), Nrf2 (**d**) and Bach1 (**e**) in undifferentiated and 4d-ATRA differentiated cells treated for 6 h with 500 μM H_2_O_2_ or 50 µM tBHQ - positive control of HO-1 induction - as indicated. Statistical analysis: n = 3 *p < 0.05 vs control; **p < 0.01 vs control; ^#^p < 0.01 vs tBHQ.

RT-PCR analysis showed a significant induction of HO-1 in both undifferentiated and 4d-ATRA differentiated cells exposed to 500 μM H_2_O_2_ or to the positive control 50 μM tBHQ for 6 h. However, while the extent of HO-1 induction is highly similar in undifferentiated cells after H_2_O_2_ or after tBHQ treatments, in 4d-ATRA differentiated cells instead, the expression of HO-1 was significantly lower after H_2_O_2_ treatment than after tBHQ exposure (Fig. 2a).

Moreover, no significant differences in the expression of the two subunits of γ-glutamyl-cisteine ligase (modulatory, GCLM and catalytic, GCLC) were observed among undifferentiated and differentiated cells exposed to H_2_O_2_ in comparison to tBHQ-treated cells or to untreated cells (Fig. 2b and c).

In addition, the mRNA levels of Nrf2 and Bach1, the two main regulators of HO-1 expression, have been analyzed in undifferentiated and differentiated cells exposed to 500 µM H_2_O_2_ or 50 µM tBHQ and no significant differences have been observed (Fig. 2d and e).

### HO-1 protein expression in response to oxidative stress occurs mainly in undifferentiated cells and favors cell survival

**Figure 3:**
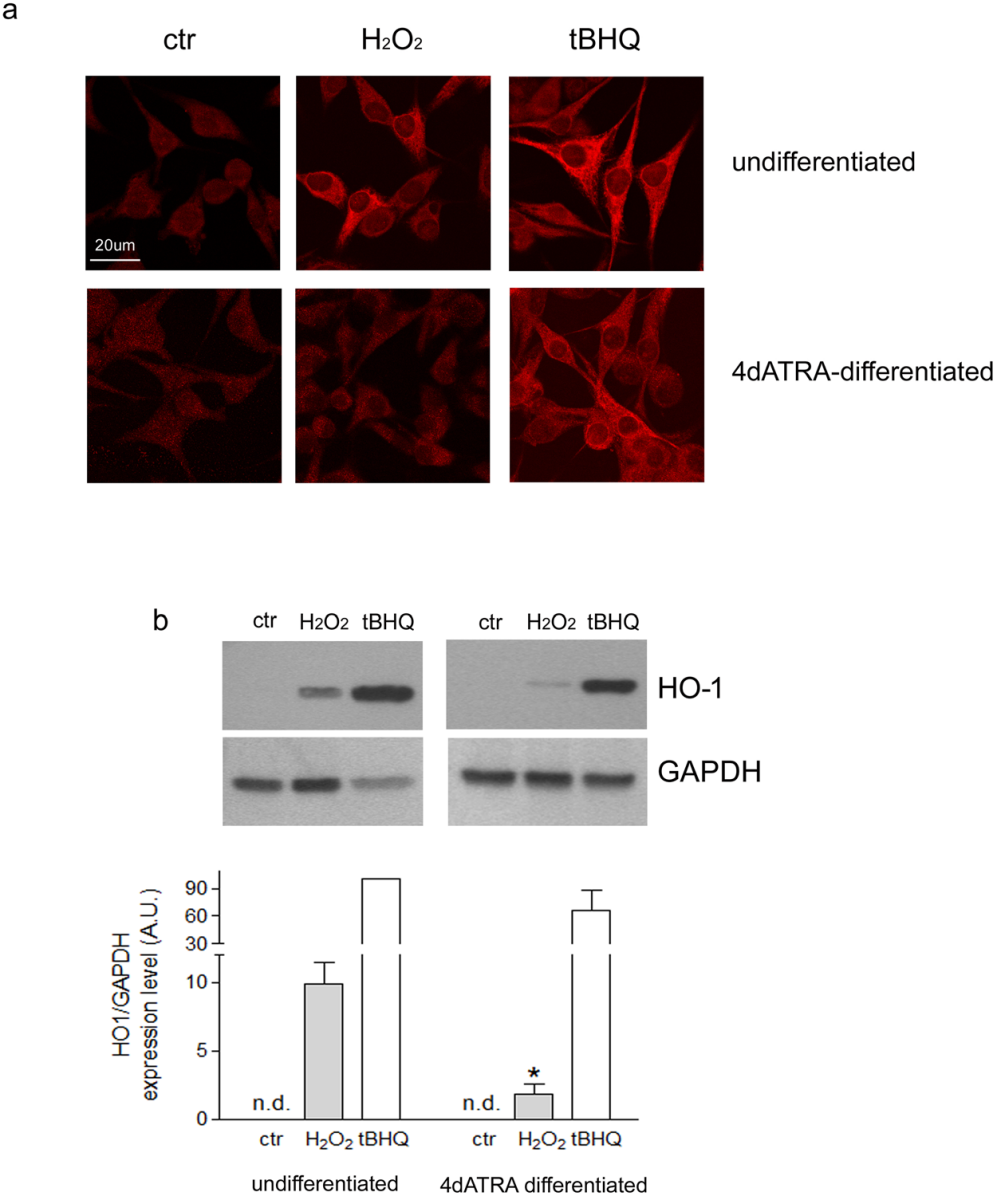
HO-1 protein expression is differently regulated in response to oxidative stress before and after differentiation. (**a**) Confocal microscopy analysis of HO-1 specific immunofluorescence in undifferentiated and 4d-ATRA differentiated cells exposed for 24 h to 500 μM H_2_O_2_ and 50 µM tBHQ, a positive control of HO-1 induction. The panels report one representative experiment of three. Scale bar: **20 μm**. (**b**) As shown by Western Blot analysis, HO-1 expression is not detectable in both undifferentiated and 4d-ATRAdifferentiated untreated cells. For this reason, the expression of HO-1 in undifferentiated cells treated with tBHQ has been considered as reference (100% of HO-1 expression). The expression of GAPHD has been used as loading control to normalize the expression of HO-1. The bands show one representative experiment of three. Statistical analysis: n = 3, *p < 0.05 vs H_2_O_2_-treated undifferentiated cells. Full-length blots are presented in supplementary information.

24 h exposure to 500 μM H_2_O_2_ clearly increased HO-1 protein expression in undifferentiated SH-SY5Y cells, as shown by confocal microscopy analysis of specific immunofluorescence. 4d-ATRA differentiated cells showed no significant HO-1 immunoreactivity in the same experimental condition. Both undifferentiated and differentiated cells were exposed to tBHQ, a positive control of HO-1 induction, which effectively increased HO-1 expression (Fig. 3a). Moreover, WB analysis confirmed that HO-1 protein level was significantly lower in 4-ATRA differentiated cells than in undifferentiated cells exposed to 500 µM H_2_O_2_ (−82% in H_2_O_2_ treated 4d-ATRA differentiated cells vs H_2_O_2_-treated undifferentiated cells). The expression of HO-1 was under the limit of detection in untreated cells and highly up-regulated by tBHQ treatment both in undifferentiated and in differentiated cells. Thus, the expression level of HO-1 in undifferentiated cells treated with tBHQ has been used as reference (100% of HO-1 expression, Fig. 3b).

**Figure 4:**
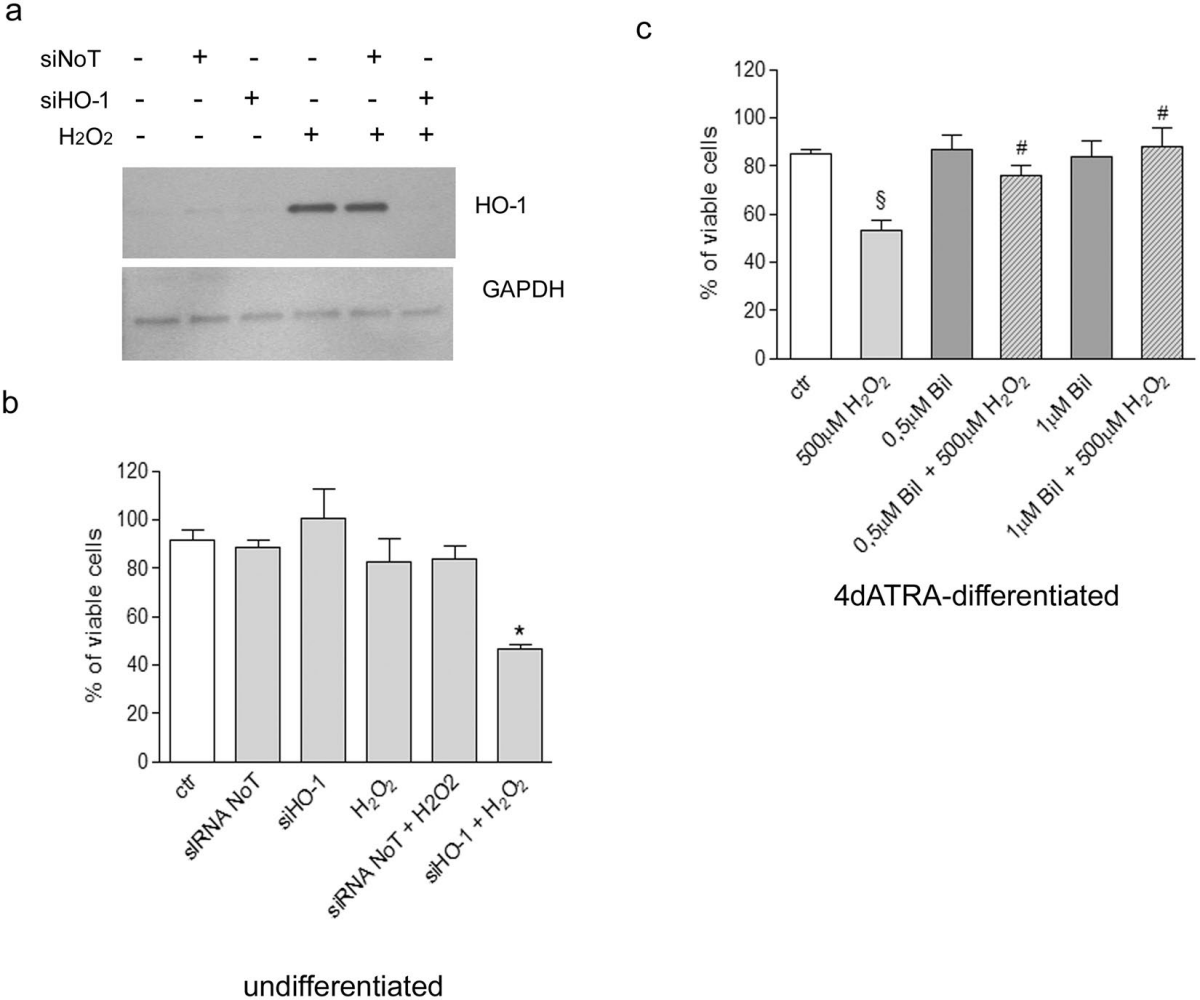
HO-1 expression is responsible for undifferentiated cell resistance to oxidative stress. Bilirubin supplementation restores viability in differentiated cells exposed to H_2_O_2_. (**a**) WB analysis of HO-1 in undifferentiated cells exposed for 24 h to 500 μM H_2_O_2_ and silenced for HO-1. GAPDH expression have been used as loading control. The bands show one representative experiment of three. (**b**) % of viability in undifferentiated cells exposed for 24 h to 500 μM H_2_O_2_ and silenced for HO-1. Some samples have been treated with a scramble siRNA (siNoT) to exclude aspecific cell responses. Statistical analysis: n = 3, *p < 0.05 vs H_2_O_2_. (**c**) 4d-ATRA differentiated cells, have been co-treated with 500 μM H_2_O_2_ and bilirubin (0.5 µM or 1 µM) in order to prevent cell death induced by cell exposure to H_2_O_2_ alone. The % of viable cells is shown. Statistical analysis: n = 3, ^§^p < 0.01 vs ctr; ^#^p < 0.05 vs H_2_O_2_. Full-length blots are presented in supplementary information.

Furthermore, HO-1 silencing, which completely abolished H_2_O_2_-dependent HO-1 up-regulation (Fig. 4a), decreased the viability of undifferentiated cells exposed to H_2_O_2_ of about 50% in comparison to untreated cells, confirming the involvement of HO-1 in undifferentiated cells resistance to oxidative stress (Fig. 4b). Then, 4d-ATRA differentiated cells supplemented with low doses of bilirubin (0.5 and 1 μM) increased resistance against H_2_O_2_, indirectly proving that the lack of HO-1-derived bilirubin was responsible for ATRA-differentiated cells sensitivity to H_2_O_2_ (Fig. 4c).

### Differentiation modifies Nrf2/Bach1 nuclear ratio in response to H_2_O_2_

HO-1 induction is regulated by the displacement of Bach1 from the ARE sequences in the two enhancers of HO-1 promoter. Bach1 displacement enables the binding of Nrf2 and the following HO-1 transcription^23^. Thus, WB analysis of Bach1 and Nrf2 expression levels has been performed in undifferentiated and differentiated cells exposed to H_2_O_2_ or tBHQ.

**Figure 5:**
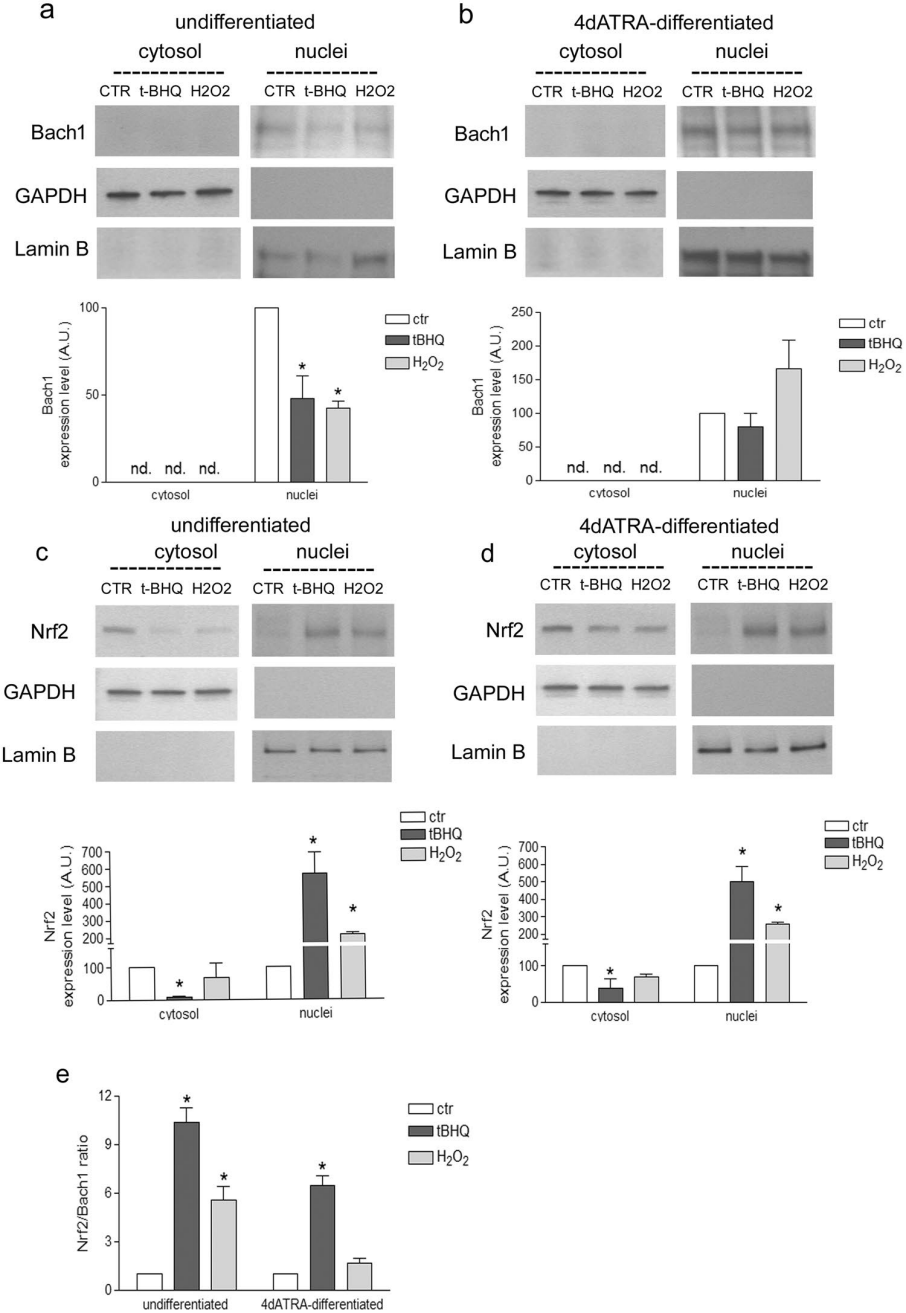
Differentiation modified the nuclear ratio between Nrf2 and Bach1 in cell treated with H_2_O_2_. (**a** and **b**) Western Blotting analysis of Bach1 cytosolic and nuclear levels of undifferentiated or 4d-ATRA differentiated cells treated for 3 h with 500 µM H_2_O_2_ or 50 µM tBHQ, as indicated. Statistical analysis: n = 3, *p < 0.05 vs ctr. (**c** and **d**) WB analysis of Nrf2 cytosolic and nuclear levels of undifferentiated or 4d-ATRA differentiated cells treated with 500 µM H_2_O_2_ or 50 µM tBHQ, as indicated. Statistical analysis: n = 3, *p < 0.05 vs ctr. The expression of GAPDH and Lamin B was checked in all the experimental conditions to verify the purity of cell fractioning and then used as loading control to normalize protein expression. (**e**) Nrf2/Bach1 ratio has been calculated to emphasize the different behavior in the two experimental conditions. Statistical analysis: n = 3, *p < 0.05 vs ctr. Full-length blots are presented in supplementary information.

Bach1 protein level was under the limit of detection in the cytosolic compartment of both undifferentiated and differentiated cells. Its nuclear level strongly decreased in undifferentiated cells treated with H_2_O_2_ in comparison to control cells (Fig. 5a). On the contrary, in 4d-ATRA differentiated cells the treatment with H_2_O_2_ did not modify Bach1 nuclear level (Fig. 5b).

The analysis of Nrf2 protein expression in cytosol and nuclei, however, revealed no differences between undifferentiated and differentiated cells. In fact, Nrf2 is mainly expressed in the cytosol in untreated cells and moves from the cytosol into the nuclei after the exposure to H_2_O_2_ in both undifferentiated (Fig. 5c) and 4d-ATRA differentiated cells (Fig. 5d). Cell have been exposed to tBHQ as positive control of Nrf2 activation. The ratio between Nrf2 and Bach1 protein levels in the nuclei has been calculated to highlight and clearly show the different behavior of differentiated cells compared with undifferentiated ones (Fig. 5e).

### Differentiation impairs Bach1 displacement form HO-1 promoter in H_2_O_2_ treated cells

**Figure 6:**
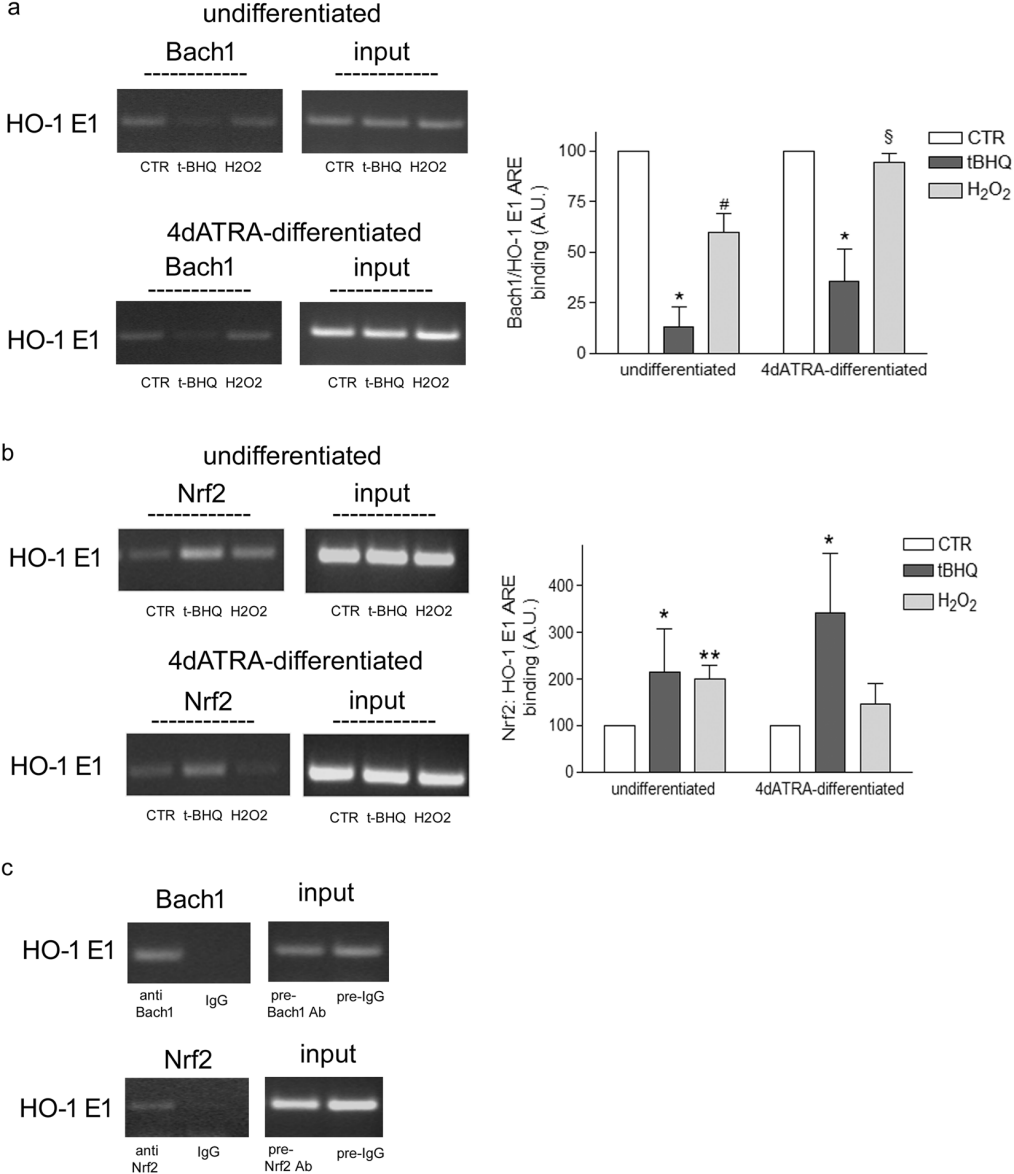
Differentiation impairs Bach1 displacement from the promoter region of HO-1 in response to H_2_O_2_. Undifferentiated and 4d-ATRA differentiated cells have been treated for 3 h with 500 μM H_2_O_2_. t-BHQ (50 μM) has been used as positive control able to displace Bach1, allowing Nrf2 to bind. (**a**) ChIP analysis of Bach1 binding to ARE sequences in the E1 promoter of HO-1. Statistical analysis: n = 3, *p < 0.01vs control; ^#^p < 0.05 vs control; ^§^p < 0.05 vs H_2_O_2_ undiff. (**b**) ChIP analysis of Nrf2 binding to ARE sequences in the E1 promoter of HO-1. Statistical analysis: n = 3, *p < 0.05 and **p < 0.01 vs control. (**c**) Negative control using IgG. As indicated no bands are detected in samples immunoprecipited with normal rabbit/goat IgG in comparison to samples immunoprecipitated by using rabbit Anti Nrf2 or goat Anti Bach1 antibodies. The intensity of PCR products amplified from immunoprecipitated samples are normalized on the intensity of bands obtained from the amplification of pre-cleared DNA (input). The bands show one representative experiment of three. Full-length gels are presented in supplementary information.

Bach1 and Nrf2 binding to ARE sequence in the enhancer 1 (E1) of HO-1 has been checked by ChIP analysis. In undifferentiated cells Bach1 binding to ARE decreased (−41, 2% vs control, Fig. 6a) and Nrf2 binding increased (+100% vs control, Fig. 6b) in response to oxidative stress, while in 4d-ATRA differentiated cells Bach1 binding did not decrease and Nrf2 binding did not significantly increase after the exposure to H_2_O_2_. tBHQ was always used as positive control able to displace Bach1 allowing Nrf2 to bind (Fig. 6). Normal IgGs have been used as control (Fig. 6c).

## Discussion

In this work we demonstrated that SH-SY5Y neuroblastoma (NB) cell differentiation induced by *all*-trans retinoic acid (ATRA) increases cell sensitivity to oxidative stress through the impairment of Bach1-depedent HO-1 induction.

Alteration of cell ability to counteract oxidative stress plays a key role not only in age-related degenerative diseases, which can be favored by the loss of adaptive responses, but also in the gain of resistance of cancer cells which progressively increase their adaptability.

We showed that undifferentiated SH-SY5Y cells are basically resistant to a medium-high degree of oxidative stress induced by cell exposure to 100–500 μM H_2_O_2_ for 24 h. However, cells differentiated by the exposure to ATRA progressively decrease viability when exposed to oxidative stress. ATRA is known to induce differentiation towards the neuronal lineage, proved by the increased expression of different neuronal markers^22^, confirmed in our experimental model as well. The acquisition of neuronal features is dependent on the generation of a controlled amount of reactive oxygen species (ROS) and the modulation of specific redox sensitive signaling pathways^5, 6^. In addition, an increased sensitivity to stress has been already demonstrated in differentiated SH-SY5Y NB cells in comparison to undifferentiated cells by our group^20^ and by others^24^. In addition, we showed that H_2_O_2_ favors the onset of apoptosis of differentiated cells, demonstrated by the expression of phosphatdylserine on the outer membrane, in agreement with other works performed in similar experimental conditions^25^.

However, in this context NB cell ability to counteract ROS generation preventing oxidative damage has not been extensively investigated.

We considered the antioxidant mechanisms regulated by Nrf2 focusing on HO-1 which are together recognized of primary importance in favoring cell survival^10^. However, among the Nrf2-dependent antioxidant responses, we also analysed the main enzymes involved in the synthesis of glutathione, namely γ-glutamyl-cisteine ligase (GCL). No differences between undifferentiated and differentiated cells exposed to oxidative stress have been observed as far as the regulation of the two subunits of GCL is concerned. In agreement with our result, the inability of neurons to up-regulate GSH synthesis has been already proved in response to nitric oxide exposure^26^. As a consequence, the level of glutathione is not modified in our experimental conditions (data not shown). Heme oxygenase 1 (HO-1), instead, is differently regulated: its expression is induced significantly more in undifferentiated cells treated with H_2_O_2_ than in differentiated cells in the same experimental conditions. Heme oxygenase (HO) is one of the most important cytoprotectant systems and its over-expression is crucial in the adaptive response to stress^27^. HO-1 and HO-2 are the two main isoforms in human cells. HO-2 is constitutively expressed in neuronal cells but it has been shown to be especially regulated in response to glucocorticoids^28^ or drugs like atorvastatin^29^. The inducible form HO-1, instead, has been shown to be up-regulated in response to ROS, heat shock, ischemia and it is also induced by its substrate heme playing a pivotal role in response to acute neuronal damage^30^ and, for this reason, we only considered HO-1. It is interesting to note that, in our experimental model, HO-1 mRNA level is detectable in control cells both before and after differentiation, while its protein level is under the limit of detection with a standard WB technique. Conceivably, even if there are still no evidence in SH-SY5Y cells, some microRNA can be involved in the regulation of HO-1 protein expression, as already shown in podocytes (miRNA 218)^31^ or in bone-marrow derived macrophages (miRNA 183)^32^. In addition, we cannot exclude that the post-transcriptional modification of HO-1 can play a crucial role in the different modulation of HO-1 expression that we observed in SH-SY5Y cells exposed to oxidative stimuli before and after differentiation, but this aspect has not been investigated yet.

In our experimental model, then, HO-1 appears to be responsible for undifferentiated cell resistance to oxidative stress. Indeed, HO-1 silencing in undifferentiated cells increases sensitivity to oxidative stress, proving the role played by HO-1 in favoring cell survival. The role of HO-1 in protecting cells from oxidative damage is ascribed to the three products of its activity, ferritin, carbon monoxide and bilirubin^33^. We showed that differentiated cells treated with low doses of bilirubin increase their resistance to oxidative stress, pointing out the lack of HO-1 derived bilirubin as a crucial mechanism of differentiated cell sensitivity to H_2_O_2_.

Bilirubin is receiving increasing attention for its powerful antioxidant activity, already recognized *in vitro* ^34^ but now well demonstrated *in vivo*, especially in the cardiovascular system^35^. Indeed, a mild increase of plasma bilirubin improves endothelial function preventing or reducing the severity of cardiovascular pathologies^36^ and metabolic diseases^37^ and, also, is protective against neuronal death induced by ischemia/reperfusion^38^. Moreover, the endogenous generation of bilirubin has been shown to be crucial in cell adaptation to oxidative stress^39^, not only in endothelial cells^40, 41^ but also in vascular muscle cells^42^ and in neuronal cells^43, 44^.

It is also important to note that bilirubin is constantly recycled in the bilirubin/biliverdin cycle by the activity of Biliverdin Reductase (BRV), allowing bilirubin to exert its antioxidant activity even at very low concentration. In fact, it has been demonstrated that 10 nM bilirubin can protect against 10000 higher concentration of hydrogen peroxide, acting complementary to GSH^45^.

Furthermore, it is worth underlining that our experimental model consists of a neuroblastoma cell line and that the endogenous generation of bilirubin from cancer cells has been recently proposed as one of the mechanisms involved in tumor progression, for instance as far as melanoma aggressiveness is concerned^46^. However, further studies are needed to understand the role of bilirubin as mediator of cancer cell survival and gain of resistance.

Next, we investigated the molecular mechanisms involved in HO-1 transcription, starting from the evaluation of its principal negative regulator Bach1. Bach1 is fundamental in the physiological adaptation to oxidative stress^23^. Through its binding to ARE sequences Bach1 represses HO-1 transcription, preventing Nrf2 binding^47^. Under stressed conditions, Bach1 is displaced from HO-1 promoter and degraded by the activity of proteasome^48^ and Nrf2 can move from the cytosol into the nucleus inducing HO-1 transcription^10^. This is completely consistent with what we have observed in undifferentiated cells exposed to oxidative stimuli.

On the contrary, in differentiated cells Bach1 is not displaced from HO-1 promoter and Nrf2 is not allowed to bind, although maintaining its ability to sense H_2_O_2_ moving into the nucleus.

In addition, in our experimental conditions, Bach1 and Nrf2 mRNA levels were not modified by oxidative stress further corroborating the hypothesis that the main regulation of both Bach1 and Nrf2 occurs at post-transcriptional level.

For the best of our knowledge, this is the first piece of evidence on the involvement of Bach1 in human neuroblastoma cell response to oxidative stress. In fact, it has been reported that Bach2 contributes to the differentiation of a murine NB cell line which, however, does not express Bach1^49^. Moreover, in the work there is no evidence concerning cell response to oxidative stress. Nonetheless, the importance of Nrf2/Bach1 ratio in the regulation of oxidative response has been highlighted in rat cortical neurons^50^. Importantly, it has also been shown that Bach1 is significantly up-regulated in the brain of subjects with Down Syndrome increasing oxidative stress and favoring the onset of Alzheimer’s disease^51^.

Thus, Bach1 might play an important role in the regulation of antioxidant responses in neuronal cells. Our work shed a new light on the involvement of retinoic acid as regulator of Bach1-dependent HO-1 induction and this is the first evidence concerning these aspects of neuronal antioxidant responses. In our experimental system, NB cells are able to differentiate toward neuronal features when treated with ATRA but this stimulus eventually impairs cell ability to counteract a new oxidative challenge. ATRA exerts its differentiating effects through the activation of its nuclear receptors RAR and RXR. It has been shown that the activation of RAR impairs Nrf2 binding to ARE^52^, but there is no evidence on a possible involvement of RAR or RXR in the modulation of Bach1 activity.

Finally, it is also important to consider the role played by different miRNA in ATRA-induced NB cell differentiation^53^ and in redox adaptation^54^ as well. Indeed, miR-155^55^ and miR-196^56^, are considerably important in the processing of Bach1 mRNA and, notably, miR-155 is dramatically reduced after retinoic acid differentiation^57^. Even though in our experimental conditions we did not find any significant modulation of Bach1 mRNA level before and after cell differentiation we cannot exclude that Bach1 stabilization on the promoter region of HO-1 is due to a modulation of other co-factors involved in Bach1 binding to ARE, for instance MafG or other related proteins. In fact, Bach1 binding to DNA is strictly dependent on its dimerization with Maf proteins^47^ and it has been recently highlighted MafG role in favoring the Bach1/DNA binding in melanoma cells, for instance^58^. Yet, the role of Maf proteins in Bach1 regulation, during neuroblastoma cell differentiation and in the response to oxidative stress is still completely unexplored. Further investigations are needed to better clarify these aspects of Bach1 regulation.

## Methods

### Cell culture, differentiation and treatments

SH-SY5Y neuroblastoma (NB) cells were cultured in RPMI 1640 medium (Euroclone, Italy) plus FBS (10%, from Euroclone), glutamine (2 mM, from Sigma-Aldrich, Italy), amphotericin B (1% Sigma-Aldrich), penicillin/streptomycin (1%, Sigma-Aldrich). Cells were split at 1:5 every 5 days and maintained in 5% CO_2_ humid atmosphere. Cells were differentiated by growth in the same medium supplemented with 10 μM all-trans retinoic acid (ATRA) (Sigma-Aldrich) for 4 and 7 days. Undifferentiated, 4 days (4d-ATRA) and 7 days (7d-ATRA) differentiated cells were exposed to increasing concentration of H_2_O_2_ (100–250–500 μM) for 24 h and the number of viable cells was measured by using Trypan Blue exclusion test and expressed as percentage. In the following experiments undifferentiated and 4d-ATRA differentiated cells were treated with 500 μM H_2_O_2_ or with 50 μM tBHQ, used as positive control of Nrf2/HO-1 induction, for 3 h (for the analysis of nuclear protein translocation and ChIP), 6 h (for the analysis of mRNA target genes) or 24 h (for the analysis of HO-1 protein expression). Some of the 4d-ATRA differentiated samples were treated with 100 nM staurosporin as a positive control of early apoptosis or exposed to 0.5 μM and 1 μM bilirubin (Sigma- Aldrich) alone or in combination with 500 μM H_2_O_2_ for 24 h.

### Evaluation of early apoptotic cells

Confocal microscopy detection of phosphatidylserine exposure on the outer membrane - marker of early apoptosis - has been performed by using the Annexin V-FITC kit (Biovision). SH-SY5Y cells were seeded on 8-well Lab-Tek II chamber slides (Nalge Nunc International) (15 × 10^3^ cells per well) and differentiated for 4 days with 10 µM retinoic acid as described before. After 24 h of treatment with 500 µM H_2_O_2_ or 100 nM staurosporin, cells were washed with PBS and incubated in the dark with Annexin V-FITC diluted 1:100 in the given binding buffer. Nuclei were counterstained with 3ng/ml To-Pro3 iodide (Invitrogen) to enable cell visualization. All the cell nuclei appear red and only early apoptotic cells show a green spotted membrane staining. Images were collected by using a three-channel TCS SP2 laser scanning confocal microscope (Leica Mycrosystems, Germany).

### HO-1 silencing

HO-1 mRNA has been silenced in undifferentiated cells exposed for 24 h to 500 μM H_2_O_2_ in 6 well plates using 120pmoles of a specific pool of oligonucleotides against human HO-1 (siHO-1, On-TargetPlus SMART pool human heme oxygenase 1; Dharmacon, USA). A scrambled pool of oligonucleotides (siRNA NoT, On-TargetPlus siControl non targeting pool; Dharmacon) has been also used to exclude aspecific cell responses. The oligonucleotides have been transfected using Polyplus - Transfection Interferin (Euroclone) as already described^59^, following manufacturer instructions.

### Extraction of RNA and Reverse Trancription-PCR

The extraction of total RNA was performed using TRIZOL (Life Techonologies, USA) by following the suggested protocol. RNA reverse transcription into cDNA was carried out by the SuperScriptTM II Reverse Transcriptase (Life-Techonologies) using random hexamer primers. cDNA amplification was achieved by using PCR Master Mix 2X (Fermentas-Dasit, Italy) and specific primers for human MAP-2, NeuroD1, GCLC, GCLM, HO-1, Nrf2, Bach1 and GAPDH. All the primer sequences used have been synthesized at Tib Mol Biol, Italy and listed in supplementary table 1. After separation on 2% agarose gel, a densitometric analysis of PCR products, stained with ethidium bromide and visualized under UV light, has been performed using a GelDoc apparatus (Bio-Rad, Italy). The expression of all the genes analyzed have been normalized on the expression of GAPDH.

### Preparation of total cell lysates and subcellular fractioning

Total protein extractions were performed using RIPA buffer while cytosolic and nuclear subcellular fractions were prepared using HEPES/EDTA buffer as previously described^60^. Protein content was measured using the BCA assay (Pierce, Thermo Scientific, USA).

### Immunoblot analysis

Proteins from total cell lysates or nuclear fractions were denatured in Laemmli buffer and then subjected to SDS-polyacrylamide gel electrophoresis (200 Volt for 50 min, Mini Protean precast TGX Gel - percentage of acrylamide is specified in supplementary information - Bio-Rad, Milan, Italy), followed by electroblotting (100 Volt for 50 min) on PVDF membrane (GE Healthcare, Amersham Place, UK). Immunodetection was performed using rabbit anti Nrf2 (1:2000, Cell Signaling), mouse anti Bach1 (1:1000, Santa Cruz Biothec) and rabbit anti HO-1 (1:2000, Origene). After incubation with specific secondary antibodies (GE Healthcare), the bands were detected by means of an enhanced chemiluminescence system (GE Healthcare). The membranes were stripped using Re-blot plus solution (Chemicon International, CA, USA) and re-probed with rabbit anti GAPDH (loading control for total lysates or cytosolic marker, 1:10000, Santa Cruz Biotech) or mouse anti lamin B (loading control for nuclear proteins, 1:1000, AbCam). Developed films were analysed using a specific software (GelDoc; Bio-Rad).

### Immunofluorescence assay

To study HO-1 expression, SH-SY5Y cells were grown as wild type or differentiated in 8-well chamber slides and then exposed to 500 μM H_2_O_2_ or 50 μM tBHQ for 24 h. By means of a standard technique of immunofluorescence (fixing in cold methanol), HO-1 expression was detected by using anti HO-1 (10 μg/ml rabbit anti HO-1, Origene) and ALEXA 633 (anti rabbit 1:400, Life-Technologies). Images were collected by using a three-channel TCS SP2 laser scanning confocal microscope (Leica Mycrosystems).

### Chromatin immunoprecipitation assay

Nrf2 and Bach1 binding to ARE sequences in the enhancer E1 in the promoter region of HO-1, was assessed by chromatin immunoprecipitation (ChIP) by using rabbit anti Nrf2 C-20 and goat anti Bach1 C-20 (Santa Cruz) antibodies, as already described^61^. Normal rabbit and goat IgG (Merk Millipore) has been employed as non specific IgG control. The sequences of the primers used for the amplification of E1 HO-1 promoter region are listed in Supplementary Table 1.

### Statistical analysis

By using GraphPad Prism software (San Diego,USA) the mean value ± SEM of the results derived from 3 or more experiments was calculated. The statistical analysis of the differences was then performed by using t-test to compare two groups or one-way ANOVA followed by Dunnett’s post-test to compare more groups.

### Data availability statement

All the data supporting this study are provided in full in the result section or as supplementary information.

## Electronic supplementary material

Supplementary information

## References

[1] Holmstrom KM, Finkel T (2014). Cellular mechanisms and physiological consequences of redox-dependent signalling. Nat Rev Mol Cell Biol.

[2] Ursini F, Maiorino M, Forman HJ (2016). Redox homeostasis: The Golden Mean of healthy living. Redox Biol.

[3] Uttara B, Singh AV, Zamboni P, Mahajan RT (2009). Oxidative stress and neurodegenerative diseases: a review of upstream and downstream antioxidant therapeutic options. Curr Neuropharmacol.

[4] Silvis AM, McCormick ML, Spitz DR, Kiningham KK (2016). Redox balance influences differentiation status of neuroblastoma in the presence of all-trans retinoic acid. Redox Biol.

[5] Nitti M (2010). PKC delta and NADPH oxidase in retinoic acid-induced neuroblastoma cell differentiation. Cell Signal.

[6] Kunzler, A. *et al*. Changes in Cell Cycle and Up-Regulation of Neuronal Markers During SH-SY5Y Neurodifferentiation by Retinoic Acid are Mediated by Reactive Species Production and Oxidative Stress. *Mol Neurobiol* (2016). 10.1007/s12035-016-0189-4 27771902

[7] Maines MD (1988). Heme oxygenase: function, multiplicity, regulatory mechanisms, and clinical applications. Faseb J.

[8] Cheng HT (2015). Ferritin heavy chain mediates the protective effect of heme oxygenase-1 against oxidative stress. Biochim Biophys Acta.

[9] Foresti, R., Green, C. J.,Motterlini, R. Generation of bile pigments by haem oxygenase: a refined cellular strategy in response to stressful insults. *Biochem Soc Symp* 177–92 (2004). 10.1042/bss0710177 15777021

[10] Loboda A, Damulewicz M, Pyza E, Jozkowicz A, Dulak J (2016). Role of Nrf2/HO-1 system in development, oxidative stress response and diseases: an evolutionarily conserved mechanism. Cell Mol Life Sci.

[11] Loboda A, Jozkowicz A, Dulak J (2015). HO-1/CO system in tumor growth, angiogenesis and metabolism - Targeting HO-1 as an anti-tumor therapy. Vascul Pharmacol.

[12] Siow RC, Sato H, Mann GE (1999). Heme oxygenase-carbon monoxide signalling pathway in atherosclerosis: anti-atherogenic actions of bilirubin and carbon monoxide?. Cardiovasc Res.

[13] Ryter SW, Otterbein LE, Morse D, Choi AM (2002). Heme oxygenase/carbon monoxide signaling pathways: regulation and functional significance. Mol Cell Biochem.

[14] Itoh K, Mimura J, Yamamoto M (2010). Discovery of the negative regulator of Nrf2, Keap1: a historical overview. Antioxid Redox Signal.

[15] Kobayashi M, Yamamoto M (2005). Molecular mechanisms activating the Nrf2-Keap1 pathway of antioxidant gene regulation. Antioxid Redox Signal.

[16] Nguyen T, Nioi P, Pickett CB (2009). The Nrf2-antioxidant response element signaling pathway and its activation by oxidative stress. J Biol Chem.

[17] Villeneuve NF, Lau A, Zhang DD (2010). Regulation of the Nrf2-Keap1 antioxidant response by the ubiquitin proteasome system: an insight into cullin-ring ubiquitin ligases. Antioxid Redox Signal.

[18] Davudian S, Mansoori B, Shajari N, Mohammadi A, Baradaran B (2016). BACH1, the master regulator gene: A novel candidate target for cancer therapy. Gene.

[19] Girvan HM, Munro AW (2013). Heme sensor proteins. J Biol Chem.

[20] Nitti M (2007). PKC delta and NADPH oxidase in AGE-induced neuronal death. Neurosci Lett.

[21] Piras S (2014). Monomeric Abeta1-42 and RAGE: key players in neuronal differentiation. Neurobiol Aging.

[22] Lopez-Carballo G, Moreno L, Masia S, Perez P, Barettino D (2002). Activation of the phosphatidylinositol 3-kinase/Akt signaling pathway by retinoic acid is required for neural differentiation of SH-SY5Y human neuroblastoma cells. J Biol Chem.

[23] Warnatz HJ (2011). The BTB and CNC homology 1 (BACH1) target genes are involved in the oxidative stress response and in control of the cell cycle. J Biol Chem.

[24] Forster JI (2016). Characterization of Differentiated SH-SY5Y as Neuronal Screening Model Reveals Increased Oxidative Vulnerability. J Biomol Screen.

[25] Ashabi G, Ahmadiani A, Abdi A, Abraki SB, Khodagholi F (2013). Time course study of Abeta formation and neurite outgrowth disruption in differentiated human neuroblastoma cells exposed to H2O2: protective role of autophagy. Toxicol In Vitro.

[26] Gegg ME (2003). Differential effect of nitric oxide on glutathione metabolism and mitochondrial function in astrocytes and neurones: implications for neuroprotection/neurodegeneration?. J Neurochem.

[27] Poon HF, Calabrese V, Scapagnini G, Butterfield DA (2004). Free radicals and brain aging. lClin Geriatr Med.

[28] Raju VS, McCoubrey WK, Maines MD (1997). Regulation of heme oxygenase-2 by glucocorticoids in neonatal rat brain: characterization of a functional glucocorticoid response element. Biochim Biophys Acta.

[29] Butterfield DA (2012). Atorvastatin treatment in a dog preclinical model of Alzheimer’s disease leads to up-regulation of haem oxygenase-1 and is associated with reduced oxidative stress in brain. Int J Neuropsychopharmacol.

[30] Chen J (2014). Heme oxygenase in neuroprotection: from mechanisms to therapeutic implications. Rev Neurosci.

[31] Yang H, Wang Q, Li S (2016). MicroRNA-218 promotes high glucose-induced apoptosis in podocytes by targeting heme oxygenase-1. Biochem Biophys Res Commun.

[32] Ke K, Sul OJ, Rajasekaran M, Choi HS (2015). MicroRNA-183 increases osteoclastogenesis by repressing heme oxygenase-1. Bone.

[33] Wegiel B, Nemeth Z, Correa-Costa M, Bulmer AC, Otterbein LE (2014). Heme oxygenase-1: a metabolic nike. Antioxid Redox Signal.

[34] Stocker R, Yamamoto Y, McDonagh AF, Glazer AN, Ames BN (1987). Bilirubin is an antioxidant of possible physiological importance. Science.

[35] Gazzin S, Vitek L, Watchko J, Shapiro SM, Tiribelli C (2016). A Novel Perspective on the Biology of Bilirubin in Health and Disease. Trends Mol Med.

[36] Maruhashi T (2012). Hyperbilirubinemia, augmentation of endothelial function, and decrease in oxidative stress in Gilbert syndrome. Circulation.

[37] Dekker D (2011). The bilirubin-increasing drug atazanavir improves endothelial function in patients with type 2 diabetes mellitus. Arterioscler Thromb Vasc Biol.

[38] Kitamura Y (2003). Hyperbilirubinemia protects against focal ischemia in rats. J Neurosci Res.

[39] Takeda TA, Mu A, Tai TT, Kitajima S, Taketani S (2015). Continuous de novo biosynthesis of haem and its rapid turnover to bilirubin are necessary for cytoprotection against cell damage. Sci Rep.

[40] He M (2015). Heme oxygenase-1-derived bilirubin protects endothelial cells against high glucose-induced damage. Free Radic Biol Med.

[41] Calay D, Mason JC (2014). The multifunctional role and therapeutic potential of HO-1 in the vascular endothelium. Antioxid Redox Signal.

[42] Clark JE, Foresti R, Green CJ, Motterlini R (2000). Dynamics of haem oxygenase-1 expression and bilirubin production in cellular protection against oxidative stress. Biochem J.

[43] Chen J, Tu Y, Moon C, Nagata E, Ronnett GV (2003). Heme oxygenase-1 and heme oxygenase-2 have distinct roles in the proliferation and survival of olfactory receptor neurons mediated by cGMP and bilirubin, respectively. J Neurochem.

[44] Chen K, Gunter K, Maines MD (2000). Neurons overexpressing heme oxygenase-1 resist oxidative stress-mediated cell death. J Neurochem.

[45] Sedlak TW (2009). Bilirubin and glutathione have complementary antioxidant and cytoprotective roles. Proc Natl Acad Sci USA.

[46] Di Biase S (2016). Fasting-Mimicking Diet Reduces HO-1 to Promote T Cell-Mediated Tumor Cytotoxicity. Cancer Cell.

[47] Zhou Y, Wu H, Zhao M, Chang C, Lu Q (2016). The Bach Family of Transcription Factors: A Comprehensive Review. Clin Rev Allergy Immunol.

[48] Su C (2016). Tetrachlorobenzoquinone induces Nrf2 activation via rapid Bach1 nuclear export/ubiquitination and JNK-P62 signaling. Toxicology.

[49] Shim KS, Rosner M, Freilinger A, Lubec G, Hengstschlager M (2006). Bach2 is involved in neuronal differentiation of N1E-115 neuroblastoma cells. Exp Cell Res.

[50] Zhang DX, Zhang LM, Zhao XC, Sun W (2017). Neuroprotective effects of erythropoietin against sevoflurane-induced neuronal apoptosis in primary rat cortical neurons involving the EPOR-Erk1/2-Nrf2/Bach1 signal pathway. Biomed Pharmacother.

[51] Di Domenico F (2015). Bach1 overexpression in Down syndrome correlates with the alteration of the HO-1/BVR-a system: insights for transition to Alzheimer’s disease. J Alzheimers Dis.

[52] Wang XJ, Hayes JD, Henderson CJ, Wolf CR (2007). Identification of retinoic acid as an inhibitor of transcription factor Nrf2 through activation of retinoic acid receptor alpha. Proc Natl Acad Sci USA.

[53] Smith B (2010). Large-scale expression analysis reveals distinct microRNA profiles at different stages of human neurodevelopment. PLoS One.

[54] Cheng X, Ku CH, Siow RC (2013). Regulation of the Nrf2 antioxidant pathway by microRNAs: New players in micromanaging redox homeostasis. Free Radic Biol Med.

[55] Pulkkinen KH, Yla-Herttuala S, Levonen AL (2011). Heme oxygenase 1 is induced by miR-155 via reduced BACH1 translation in endothelial cells. Free Radic Biol Med.

[56] Go H (2016). MiR-196a regulates heme oxygenase-1 by silencing Bach1 in the neonatal mouse lung. Am J Physiol Lung Cell Mol Physiol.

[57] Culpan D, Kehoe PG, Love S (2011). Tumour necrosis factor-alpha (TNF-alpha) and miRNA expression in frontal and temporal neocortex in Alzheimer’s disease and the effect of TNF-alpha on miRNA expression *in vitro*. Int J Mol Epidemiol Genet.

[58] Fang M, Hutchinson L, Deng A, Green MR (2016). Common BRAF(V600E)-directed pathway mediates widespread epigenetic silencing in colorectal cancer and melanoma. Proc Natl Acad Sci USA.

[59] Furfaro AL (2012). Resistance of neuroblastoma GI-ME-N cell line to glutathione depletion involves Nrf2 and heme oxygenase-1. Free Radic Biol Med.

[60] Furfaro AL (2012). Impaired synthesis contributes to diabetes-induced decrease in liver glutathione. Int J Mol Med.

[61] Furfaro AL (2016). Role of Nrf2, HO-1 and GSH in Neuroblastoma Cell Resistance to Bortezomib. PLoS One.

