# Supplementary material for: Differentiation impairs Bach1 dependent HO-1 activation and increases sensitivity to oxidative stress in SH-SY5Y neuroblastoma cells
Source: Sci Rep. 2017 Aug 8;7:7568. doi: 10.1038/s41598-017-08095-7 (PMC5548785; doi:10.1038/s41598-017-08095-7)
Supplement: Supplementary file 1 — Supplementary information [file 41598_2017_8095_MOESM1_ESM.pdf]

Supplementary information

Manuscript SREP -17-11560A

Title

Differentiation impairs Bach1 dependent HO-1 activation and increases sensitivity to oxidative stress in SH-SY5Y neuroblastoma cells.

Authors

Sabrina Piras[1], Anna Lisa Furfaro[2], Lorenzo Brondolo[1], Mario Passalacqua[1], Umberto Maria Marinari[1], Maria Adelaide Pronzato[1], Mariapaola Nitti[1]

1 Department of Experimental Medicine, University of Genoa, Via L.B. Alberti 2, 16132, Genoa, Italy

2 Giannina Gaslini Institute, Via Gerolamo Gaslini 5, 16147, Genoa, Italy

Supplementary Fig.1

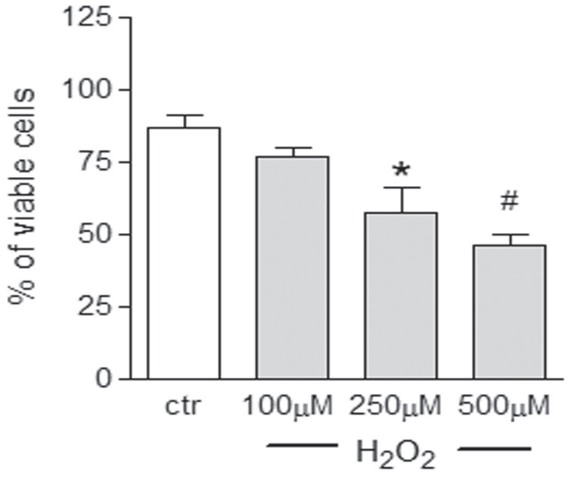

7dATRA-differentiated

Supplementary Tab.1. Primer sequences used

Name	Primer Fw 5'-3'	Primer Rv 5'-3'	Size
MAP-2	tca gag gca atg acc tta cc	gtg gta ggc tct tgg tct tt	320 bp
NeuroD1	ccc ttc ctt tga tgg acc cc	aaa tgg tga aac tgg cgt gc	296 bp
HO-1	gtc caa cat cca gct ctt tga gg	gac aaa gtt cat ggc cct ggg a	284 bp
GCLM	cca gat gtc ttg gaa tgc	tgc agt caa atc tgg tgg	408 bp
GCLC	atg gag gtg caa tta aca gac	act gca ttg cca cct ttg ca	206 bp
Nrf2	cct gag tta cag tgt ctt aa	act gag tgt tct ggt gat g	499 bp
Bach1	tgc gat gtc acc atc ttt gt	ggt ctg cag tgg agt cca aa	310 bp
GAPDH	gtc ttc acca cc atg gag aa	atc cac agt ctt ctg ggt gg	266 bp
HO-1 E1	gct gcc caa acc act tct gt	gcc ctt tca cct ccc acc ta	200 bp

Fig. 3b Full-length blots

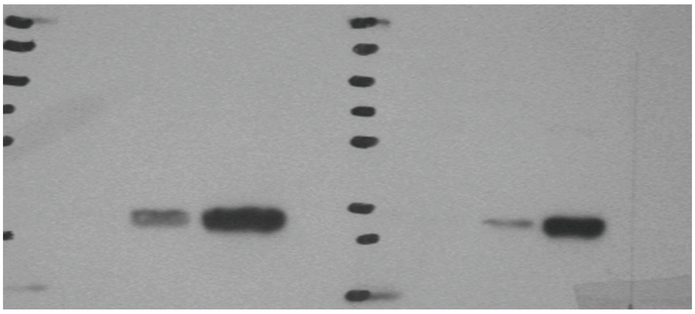

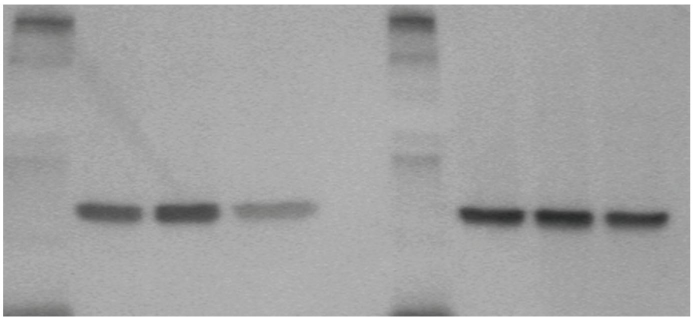

Fig. 4a Full-length blots

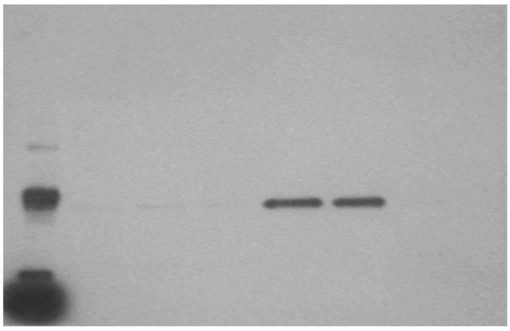

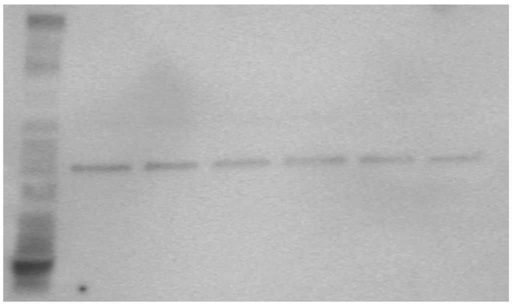

Fig. 5a and b, Full-length blots

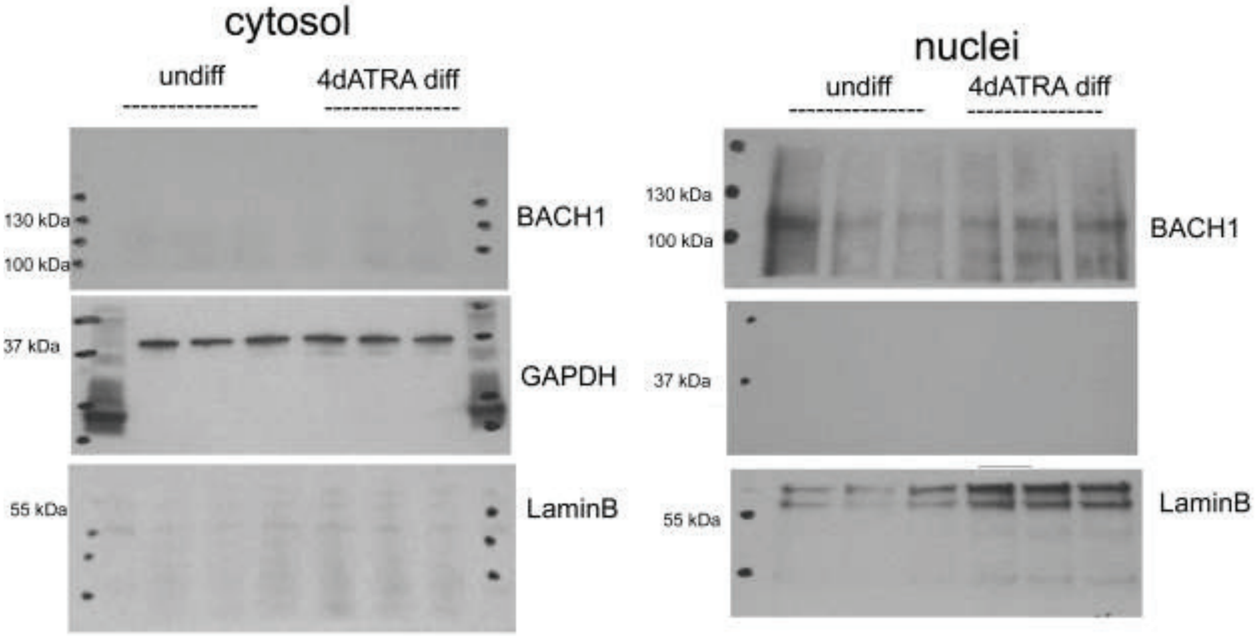

Fig. 5c and d, Full-length blots

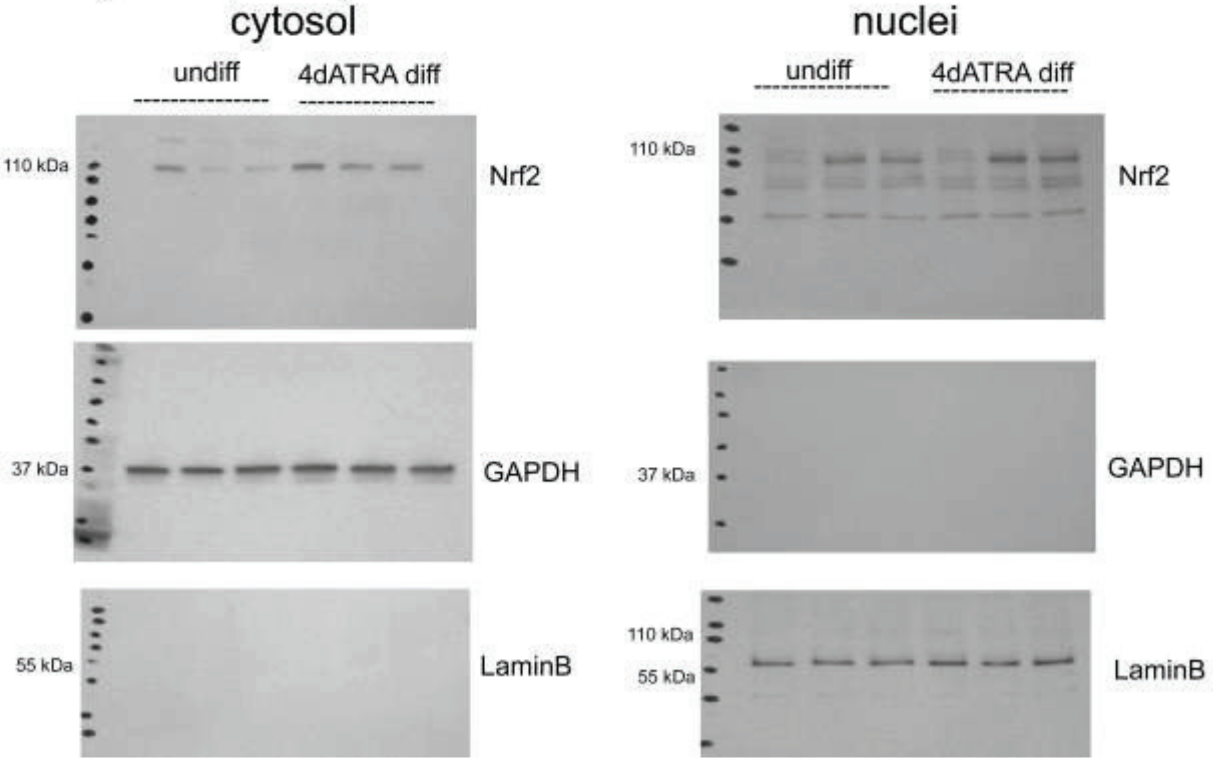

Fig. 6 Full-length gels

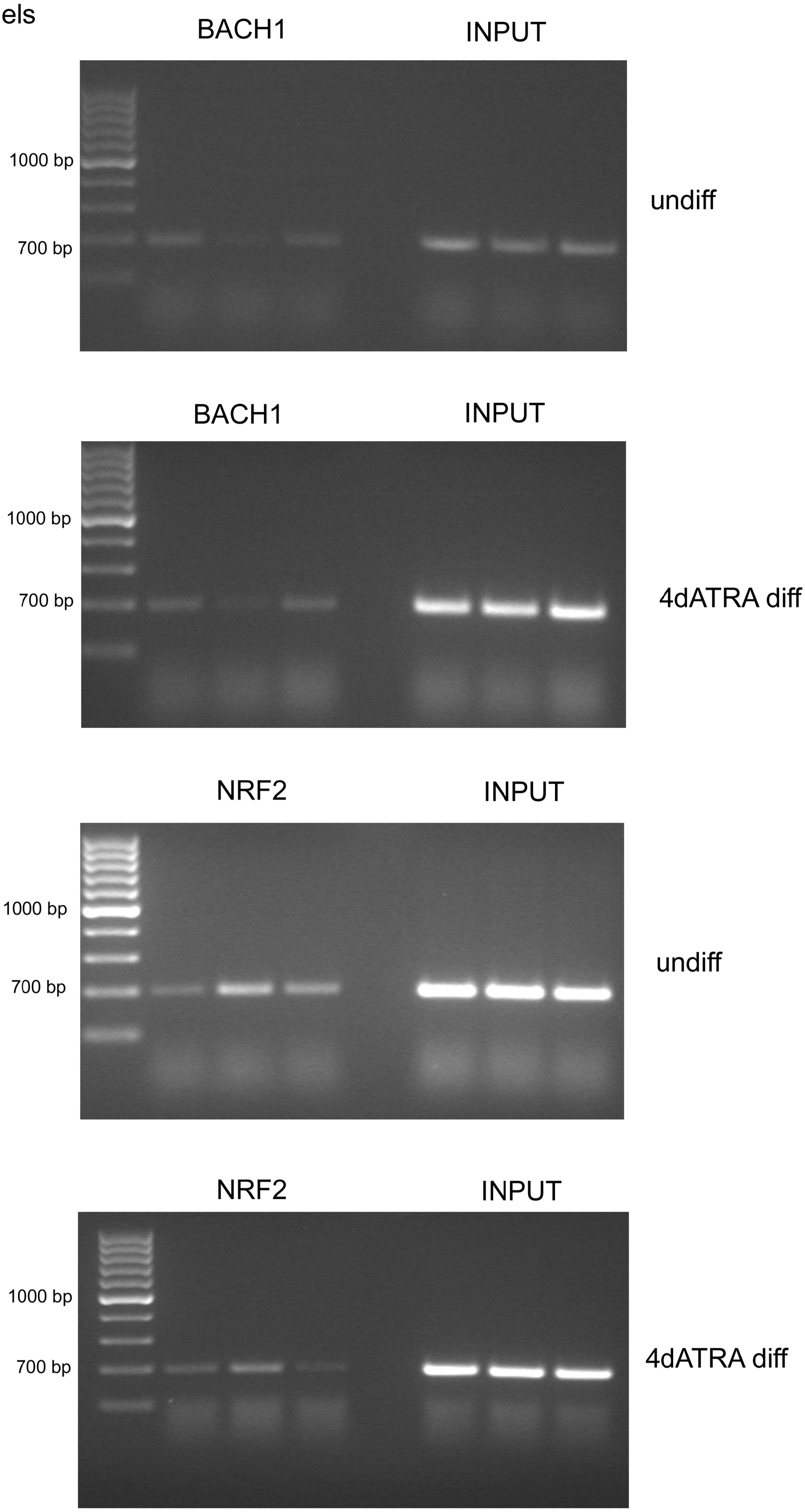

Fig. 6c Full-length gels

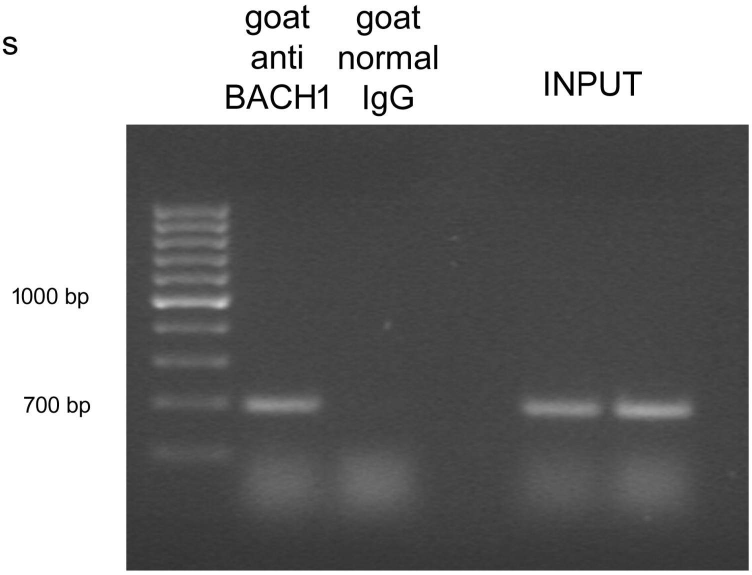

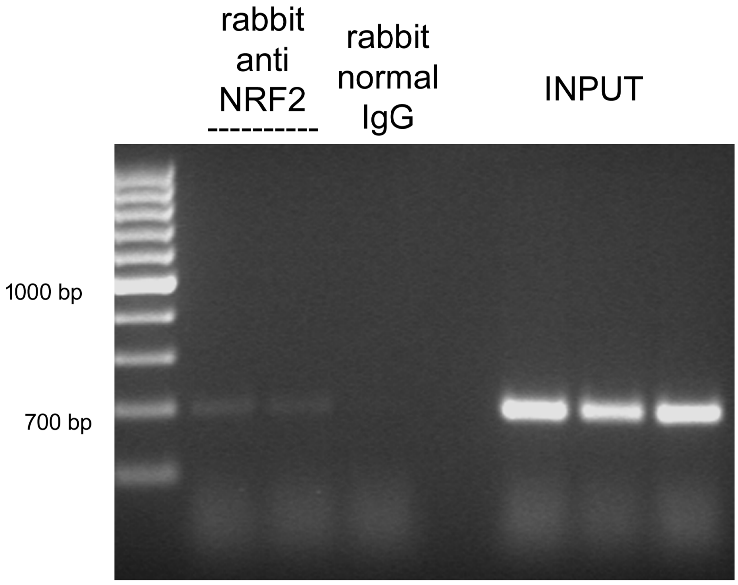

Legends of Supplementary Information

Supplementary Fig.1

Viability test (Trypan blue) 7dATRA-differentiated cells treated 24h with increasing concentrations of H_2O_2 (100µM, 250µM and 500µM). Viability is decreased about 52% and 54% respectively at the highest doses of H_2O_2. Statistical analysis: n=3, * p<0.05 vs ctr; # p<0.01vs ctr.

Supplementatry Tab.1

Primer sequences used for the amplification of MAP2, NeuroD1, HO-1, GCLC, GCLM, Nrf2, Bach1 and GAPDH by RT-PCR analysis and for the amplification of the enhancer 1 in the promoter region of HO-1 (HO-1 E1) by ChIP

Fig.3b Full-length blots

10% precast gels have been used to analyse HO-1 expression. Membranes have been stripped and reprobed for GAPDH. Molecular weights are indicated.

Fig.4a Full-length blots

4-20% precast gradient gels have been used to analyse HO-1 expression. Membranes have been stripped and reprobed for GAPDH. Molecular weights are indicated.

Fig.5a and b Full-length blots

7.5% precast gels have been used to analyse cytosolic and nuclear Bach1 expression. In order to save antibody, the membranes were cut and the upper part have been probed with anti Bach1 ab while the lower parts with anti GAPDH or anti lamin B abs to check the purity of cell fractioning and to normalize. Molecular weights are indicated.

Fig.5c and d Full-length blots

10% precast gels have been used to analyse cytosolic and nuclear Nrf2 expression. The main band (110kDa) has been considered as the only one modulated by tBHQ treatment. Membranes have been stripped and reprobed with anti GAPDH or anti lamin B abs to check the purity of cell fractioning and to normalize. Molecular weights are indicated.

Fig.6 Full-length gels

The amplification products of Bach1- and Nrf2-ARE binding site on E1 HO-1 promoter were analyzed in a 2% agarose gel stained with ethidium bromide and visualized under UV light. Molecular weights are indicated.
